# Insecticide Resistance Profiles and Synergism of Field *Aedes aegypti* from Indonesia

**DOI:** 10.1371/journal.pntd.0010501

**Published:** 2022-06-06

**Authors:** Christina Natalina Silalahi, Wu-Chun Tu, Niann-Tai Chang, G. Veera Singham, Intan Ahmad, Kok-Boon Neoh

**Affiliations:** 1 Department of Entomology, National Chung Hsing University, Taichung, Taiwan; 2 Department of Plant Medicine, National Pingtung University of Science and Technology, Pingtung, Taiwan; 3 Centre for Chemical Biology, Universiti Sains Malaysia, Bayan Lepas, Penang, Malaysia; 4 School of Life Sciences and Technology, Institut Teknologi Bandung, Bandung, West Java, Indonesia; Centers for Disease Control and Prevention, Puerto Rico, UNITED STATES

## Abstract

Information on the insecticide resistance profiles of *Aedes aegypti* in Indonesia is fragmentary because of the lack of wide-area insecticide resistance surveillance. We collected *Ae*. *aegypti* from 32 districts and regencies in 27 Indonesian provinces and used WHO bioassays to evaluate their resistance to deltamethrin, permethrin, bendiocarb, and pirimiphos-methyl. To determine the possible resistance mechanisms of *Ae*. *aegypti*, synergism tests were conducted using piperonyl butoxide (PBO) and S,S,S-tributylphosphorotrithioates (DEF). The *Ae*. *aegypti* from all locations exhibited various levels of resistance to pyrethroids. Their resistance ratio (RR_50_) to permethrin and deltamethrin ranged from 4.08× to 127× and from 4.37× to 72.20×, respectively. In contrast with the findings of other studies, most strains from the highly urbanized cities on the island of Java (i.e., Banten, Jakarta, Bandung, Semarang, Yogyakarta, and Surabaya) exhibited low to moderate resistance to pyrethroids. By contrast, the strains collected from the less populated Kalimantan region exhibited very high resistance to pyrethroids. The possible reasons are discussed herein. Low levels of resistance to bendiocarb (RR_50_, 1.24–6.46×) and pirimiphos-methyl (RR_50_, 1.01–2.70×) were observed in all tested strains, regardless of locality. PBO and DEF synergists significantly increased the susceptibility of *Ae*. *aegypti* to permethrin and deltamethrin and reduced their resistance ratio to less than 16×. The synergism tests suggested the major involvement of cytochrome P450 monooxygenases and esterases in conferring pyrethroid resistance. On the basis of our results, we proposed a 6-month rotation of insecticides (deltamethrin + synergists ➝ bendiocarb ➝ permethrin + synergists ➝ pirimiphos-methyl) and the use of an insecticide mixture containing pyrethroid and pyrimiphos-methyl to control *Ae*. *aegypti* populations and overcome the challenge of widespread *Ae*. *aegypti* resistance to pyrethroid in Indonesia.

## Author summary

Insecticide resistance is a major impediment to the successful management of vector-transmitted diseases because it increases the vector’s chances of surviving under insecticide treatment. In Indonesia, the implementation of insecticide resistance management at the national level is particularly challenging due to the vast area and regional disparities in terms of population, health, and socioeconomic status. Previous studies on determining insecticide resistance of *Aedes* mosquito only focused on several cities in some provinces of Indonesia, making resistance monitoring results difficult to interpret and arguably reflect the generality in Indonesia. To complicate the matter, data released by the Ministry of Agriculture of Indonesia in 2022 showed that approximately 82% of insecticides registered to control *Ae*. *aegypti* in Indonesia are pyrethroid-based products. Principally, we found that the synergists PBO and DEF significantly reduce the resistance of field *Ae*. *aegypti* from Indonesia toward permethrin and deltamethrin. Bendiocarb and pirimiphos-methyl remain highly toxic to the field strains of *Ae*. *aegypti*. We suggest the feasible choice of insecticide group for *Ae*. *aegypti* vector management based on the currently registered insecticide inventory. The finding also underscores the urgent need to approve other non-pyrethroid-based insecticides as alternative tools for reducing the risk of resistance development during an outbreak.

## Introduction

*Aedes aegypti* is the primary vector of dengue virus (DENV), which causes dengue fever. The disease is becoming a major public health concern in Indonesia [1,2]. Dengue cases were first documented in the Special Capital Region of Jakarta and Surabaya (East Java) in 1968, but cases are now reported in almost all regions of the 34 provinces of Indonesia. In 2019, dengue cases were reported in 481 out of 514 regencies and cities, accounting for 93.58% of the regencies and cities in the 34 provinces; by contrast, cases were reported in only 85.60% of the regencies and cities in 2018 [3]. The Ministry of Health of Indonesia reported that the incidence rate (IR) of dengue fever in Indonesia increased from 24.75 per 100,000 in 2018 to 51.48 per 100,000 in 2019 [3].

Because the availability of effective dengue vaccines is still limited, vector control is regarded as a strategy for preventing and reducing DENV transmission. In Indonesia, various dengue vector control programs are implemented, including environmental management and chemical control [4–6]. Health authorities have emphasized the role of community involvement in reducing the number of potential mosquito breeding sites through the 3M campaign (i.e., covering and cleaning potential breeding spots and burying discarded containers) [5]. However, the mosquito population in Indonesia remains high [7]. *Ae*. *aegypti* control in Indonesia is still mainly implemented through the application of chemical insecticides, especially during an outbreak.

Four main classes of insecticides are used to control mosquitoes, namely organochlorines, organophosphates, pyrethroids, and carbamates [8]. The organochlorine insecticides DDT and dieldrin were introduced for malaria vector control in Indonesia in the 1950s. However, DDT and dieldrin use was suspended in the 1970s because mosquitoes developed a resistance to DDT and dieldrin [9]. Since the 1970s, organophosphates (malathion and temephos) have been widely used in Indonesia to suppress mosquito vectors. Subsequently, pyrethroids came into use in the 1980s and were used in agricultural sectors and nationwide mosquito control programs in Indonesia [6,10,11]. The extensive use of pyrethroids in past decades has led to the development of pyrethroid resistance in *Ae*. *aegypti*, which has become a challenge for dengue vector control programs and affected the efficacy of insecticide-based vector control strategies [12,13].

Two common pyrethroid resistance mechanisms in *Ae*. *aegypti* are target-site and metabolic resistance [6,14]. Target-site resistance is caused by single or multiple mutations in the sequence of a target protein, inducing insensitivity at the target site of an insecticide [15,16]. The point mutations at the voltage-gated sodium channel (VGSC), which are referred to as knockdown-resistance (*kdr*), have been reported in pyrethroid-resistant populations in various Indonesian cities, including Jakarta [11], Magelang (Central Java) [17], Semarang (Central Java) [18,19], Yogyakarta [6], Denpasar (Bali) [20], Makassar (South Sulawesi) [21], Banjarmasin (South Kalimantan) [4], Belu and Ende (East Nusa Tenggara) [22], and Palu (Central Sulawesi) [22]. Metabolic resistance, which increases detoxifying enzyme levels, enables an insecticide to be degraded before it reaches its target [6,23]. This form of resistance is associated with three groups of enzymes, namely glutathione S-transferases, esterases, and cytochrome P450 monooxygenases [8,16]. In 2006, resistance to permethrin and deltamethrin was documented in the Bandung (West Java) strain and reported to be related to increased oxidative and hydrolytic activities [10]. Widiastuti et al. observed that the metabolic enzymes of esterase and cytochrome P450 monooxygenases confer *Ae*. *aegypti* populations from Wonosobo (Central Java) with resistance to malathion and cypermethrin, resulting in low mortality rates of 23.30% and 46.7%, respectively [24]. However, information on the resistance profiles of *Ae*. *aegypti* is fragmentary because most studies have focused on a small number of highly urbanized cities. Therefore, these studies have not determined the overall resistance of *Ae*. *aegypti*, which is required to formulate a nationwide chemical control strategy for dengue vector mitigation.

We surveyed 32 strains of *Ae*. *aegypti* from 27 provinces in Indonesia to expand on these findings. Collecting data through nationwide evaluations is a laborious task; thus, such data are rarely available. The resistance levels of *Ae*. *aegypti* to several commonly used insecticides (namely deltamethrin [type II pyrethroid group], permethrin [type I pyrethroid group], bendiocarb [carbamate group], and pirimiphos-methyl [organophosphate group]) were measured using the WHO insecticide susceptibility test. Synergism tests involving the use of the synergists piperonyl butoxide (PBO) and S,S,S-tributylphosphorotrithioates (DEF) were conducted to determine possible resistance mechanisms. PBO inhibits the activity of mixed-function oxidases, which are also known as cytochrome P450 [25–27], whereas DEF is the inhibitor of esterases [26]. If the susceptibility of the tested mosquitoes is irresponsive to both PBO and DEF, other mechanisms such as *kdr* mutation may be implicated. Primarily, these results provide valuable information on the selection of insecticides and synergists at operational levels, and they can be used as a basis for the development of a strategic insecticide resistance management framework for dengue vector control in Indonesia.

## Materials and methods

### Mosquito samples

**Fig 1:**
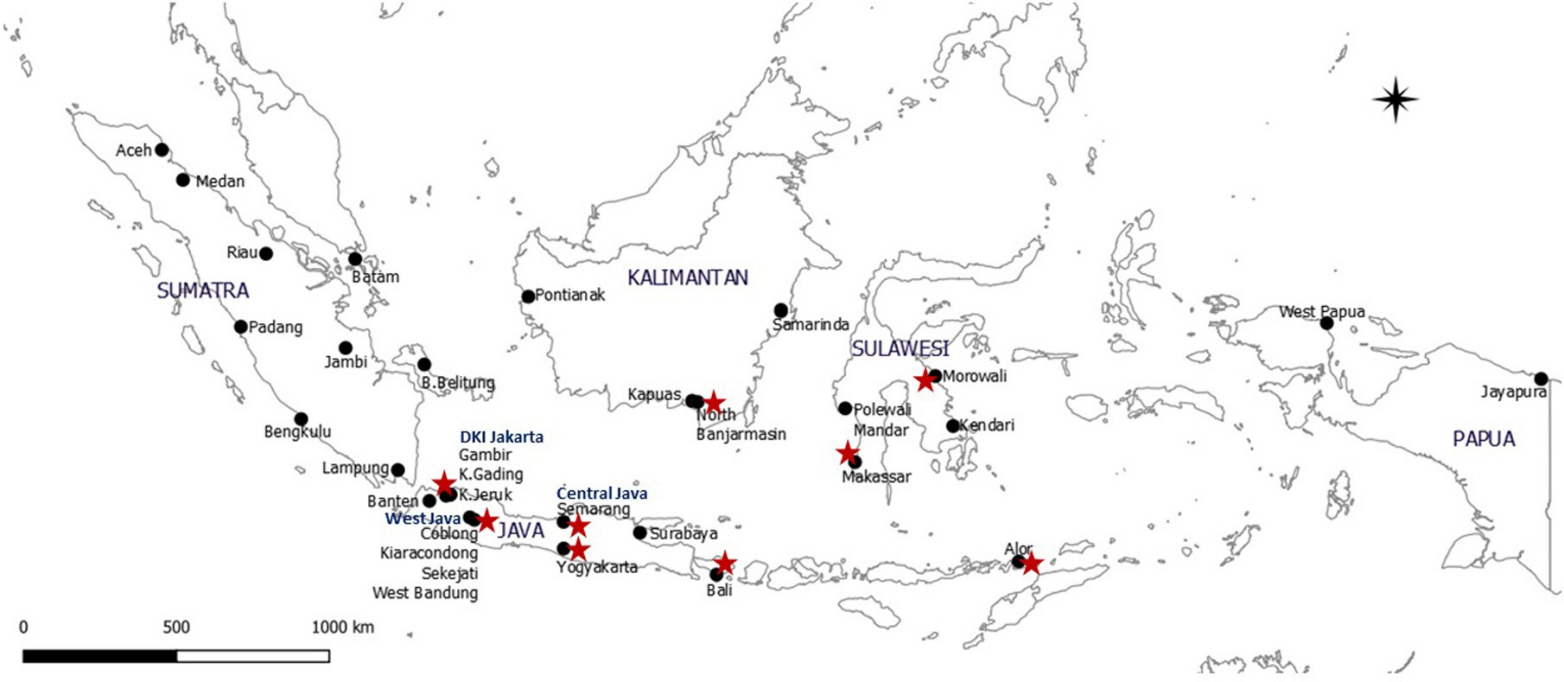
Map of *Ae*. *aegypti* collection sites in Indonesia (32 strains). Map base layer was obtained from https://www.naturalearthdata.com/. Map base layer was modified in QGIS software version 3.4 Madeira. Note: ★ indicates sites where pyrethroid-resistant *Ae*. *aegypti* mosquitoes were previously reported.

*Ae*. *aegypti* eggs were collected using ovitraps from 32 districts and regencies (Fig 1 and S1 Table) in 27 provinces of Indonesia between May and December 2019. The collection sites were randomly selected on the basis of data from the Ministry of Health of Indonesia, which reported dengue hemorrhagic fever cases in 85.60% of the districts and regencies of the 34 Indonesian provinces in 2018 [3]. Three to five ovitraps were installed at each collection site. Each ovitrap consisted of a black plastic container with a 10.5-cm diameter, a 13-cm height, and a lid with a circular aperture in the middle (4-cm diameter). A paper towel (22.5 cm × 22.7 cm), which was used as an oviposition strip, was divided into two segments and positioned in each ovitrap. Each ovitrap was then filled with water to a capacity of 50%–60% to collect mosquito eggs. The ovitraps were then placed in residential areas with the oral consent of local residents. Overall, approximately 20 to 100% of the traps installed at a collection site were positive for the presence of *Aedes* mosquito eggs.

### Mosquito rearing

Adult *Ae*. *aegypti* mosquitoes were reared in a laboratory at 25 ± 1°C, 65% ± 5% relative humidity, and a photoperiod of 12:12 (L:D) h. The mosquito rearing methods were based on those described by Bong et al. [28]. Field-collected eggs of *Ae*. *aegypti* were immersed in dechlorinated water with two to three drops of Vitamin C (1 mL) for 24 h to induce hatching. Approximately 200–250 larvae were placed in 1 L of dechlorinated water in a plastic container (29.5 cm [length] × 23.0 cm [width] × 5.0 cm [height]). The larvae were subjected to an artificial larvae diet ad libitum, which consisted of pork liver powder and yeast at a ratio of 1:1. Subsequently, the adult *Ae*. *aegypti* mosquitoes that emerged from the field-collected eggs were provided with a constant supply of 10% sucrose solution. Insecticide susceptibility tests were conducted using F2–F4 progenies emerging from field-collected eggs, and the Bora-Bora strain (F34–F35) was used as an insecticide susceptibility reference strain.

### Insecticide susceptibility test

Insecticide bioassays were conducted using a WHO test kit to determine the susceptibility of the *Ae*. *aegypti* field strains. A batch of 20–25 unmated, non-blood-fed *Ae*. *aegypti* females aged 5–7 days was transferred to four plastic holding tubes (hence, four replications were performed) lined with insecticide-impregnated filter paper (Whatman grade 1, 12 cm × 15 cm). The females were starved for 6 h prior to testing. The filter papers were impregnated using the insecticides with test concentrations based on diagnostic dosages for *Ae*. *aegypti* [26], namely 0.566% permethrin (type I pyrethroid group), 0.014% deltamethrin (type II pyrethroid group), and 0.212% pirimiphos-methyl (organophosphate group). Subsequently, 0.1% bendiocarb (carbamate group), in accordance with the WHO guidelines for the diagnostic dosage for anopheline mosquitoes, was used [29]. For the control, mosquitoes were exposed to paper that was only impregnated with carrier oil and acetone. The number of knockdown mosquitoes was constantly recorded over time until a minimum knockdown of 80% was achieved to ensure the mortality in probits could be fitted satisfactorily.

### Synergism tests

Synergist bioassays were conducted to assess the effectiveness of synergists in detoxifying insecticides. The tests were performed by exposing the mosquitoes to filter paper treated with sublethal doses of 4% w/w piperonyl butoxide (PBO) and 5% w/w S,S,S-tributyl phosphorotrithioate (DEF) [26]. Adult mosquitoes were exposed to each synergist for 1 h before exposure to the test insecticide.

### Data analysis

The data from insecticide bioassays were subjected to probit analysis by using SPSS analysis version 11.0 (SPSS Inc., Chicago, IL) to obtain a 50% knockdown time (KT_50_) value. To determine the susceptibility of the *Ae*. *aegypti* field strains, the level of insecticide resistance was categorized by resistance ratio (RR). RR_50_ was calculated by dividing the KT_50_ value of the field strain *Ae*. *aegypti* by that of the Bora-Bora reference strain [26]. The synergist ratio (SR_50_) was then calculated by dividing the KT_50_ of the field strain *Ae*. *aegypti* exposed to insecticide alone with that exposed to the insecticide and synergist [30]. RR_50_ values were classified in accordance with the criteria provided by Koou et al. [26], which are as follows: susceptible (RR_50_, < 1×), low resistance (RR_50_, 1–10×), moderate resistance (RR_50_, 11–30×), high resistance (RR_50_, 31–100×), and very high resistance (RR_50_, > 100×).

## Results

### Insecticide susceptibility status

#### Pyrethroids

**Fig 2:**
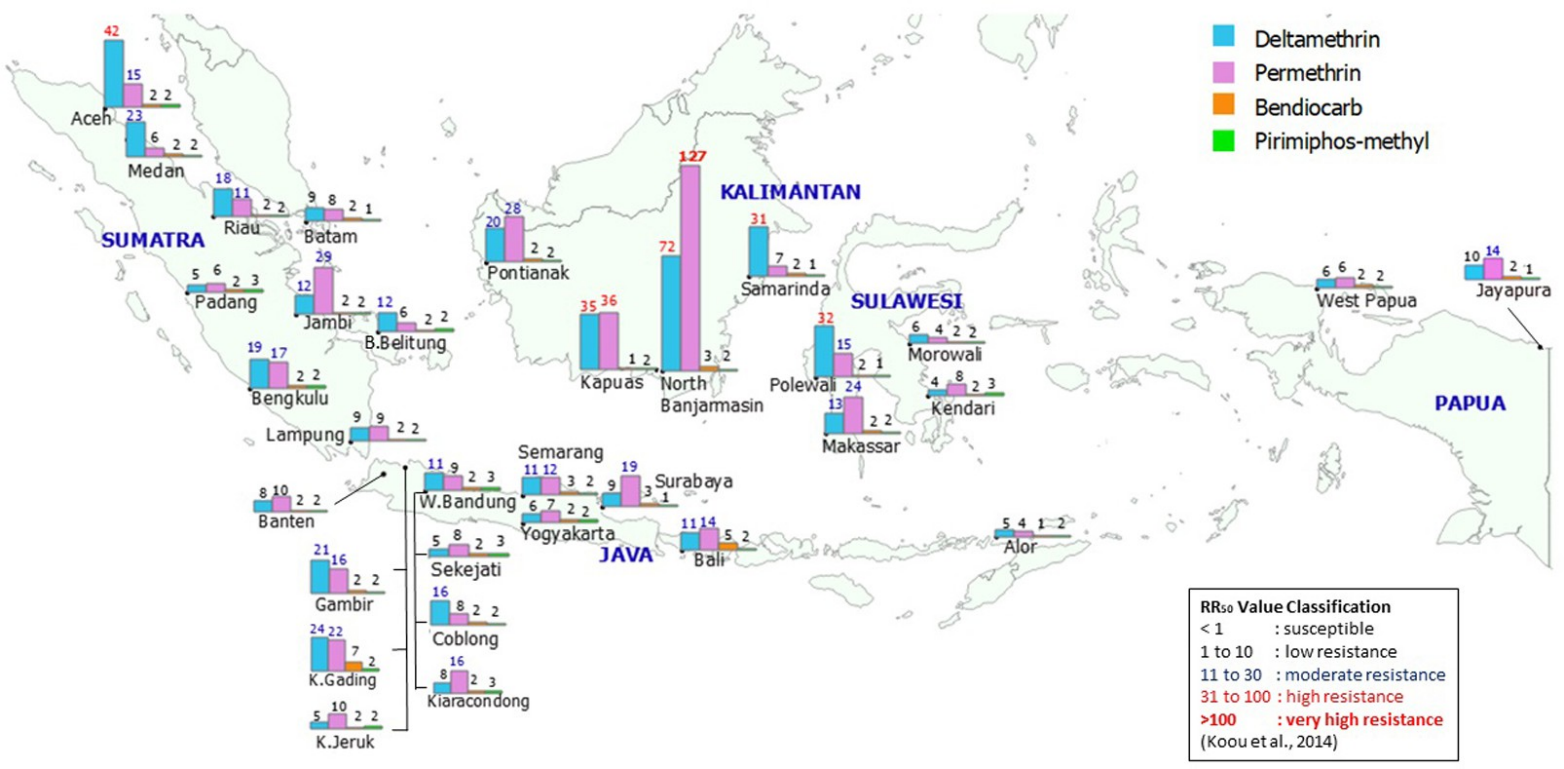
Map of susceptibility of *Ae*. *aegypti* field strains to tested insecticides. Map base layer was obtained from https://www.naturalearthdata.com/. Map base layer was modified in QGIS software version 3.4 Madeira. Note: The number above each bar represents RR_50_, which was calculated relative to the susceptible reference strain (Bora-Bora).

All 32 field strains of *Ae*. *aegypti* collected from the 27 provinces exhibited various levels of resistance to both deltamethrin and permethrin (Fig 2 and S2 and S3 Tables). In particular, the North Banjarmasin strain from South Kalimantan province exhibited high resistance to deltamethrin (RR_50_ = 72.20×). The mosquitoes from other Kalimantan areas (Kapuas and Samarinda), West Sulawesi (Polewali Mandar), and Aceh (Langsa) also exhibited high deltamethrin resistance (RR_50_ = 30.76×–42.06×). Similarly, for permethrin, the *Ae*. *aegypti* from North Banjarmasin exhibited very high resistance compared with other field strains (RR_50_ = 127.00×). A high resistance to permethrin was also observed in the *Ae*. *aegypti* from Kapuas (RR_50_ = 35.52×). However, the *Ae*. *aegypti* from Samarinda exhibited low resistance to permethrin (RR_50_ = 7.25×). The field strains from Aceh and Polewali Mandar both exhibited moderate resistance to permethrin, with RR_50_ values of 15.12× and 14.97×, respectively. Notably, six cities on Java Island demonstrated low to moderate resistance to deltamethrin and permethrin; the RR_50_ values with respect to deltamethrin and permethrin, respectively, were as follows: 8.08× and 9.98× for Banten, 21.15× and 15.64× for Gambir (in Jakarta), 5.11× and 10.07× for Kebon Jeruk (in Jakarta), 24.35× and 22.33× for Kelapa Gading (in Jakarta), 11.05× and 9.17× for West Bandung (in Bandung); 7.45× and 16.06× for Kiaracondong (in Bandung), 16.19× and 8.10× for Coblong (in Bandung), 5.06× and 7.90× for Sekejati (in Bandung), 10.98× and 11.62× for Semarang, 5.87× and 7.27× for Yogyakarta, and 8.58× and 19.39× for Surabaya.

#### Organophosphate and carbamate

All 32 field-collected *Ae*. *aegypti* strains exhibited low resistance to both pirimiphos-methyl and bendiocarb (Fig 2 and S4 and S5 Tables). The RR_50_ values of the 32 field strains with respect to resistance to bendiocarb were between 1.24× and 6.46× higher than those of the lab strain. In addition, all field strains had no or low resistance to pirimiphos-methyl, with RR_50_ values between 1.01× and 2.70×.

### Synergist assays

**Fig 3:**
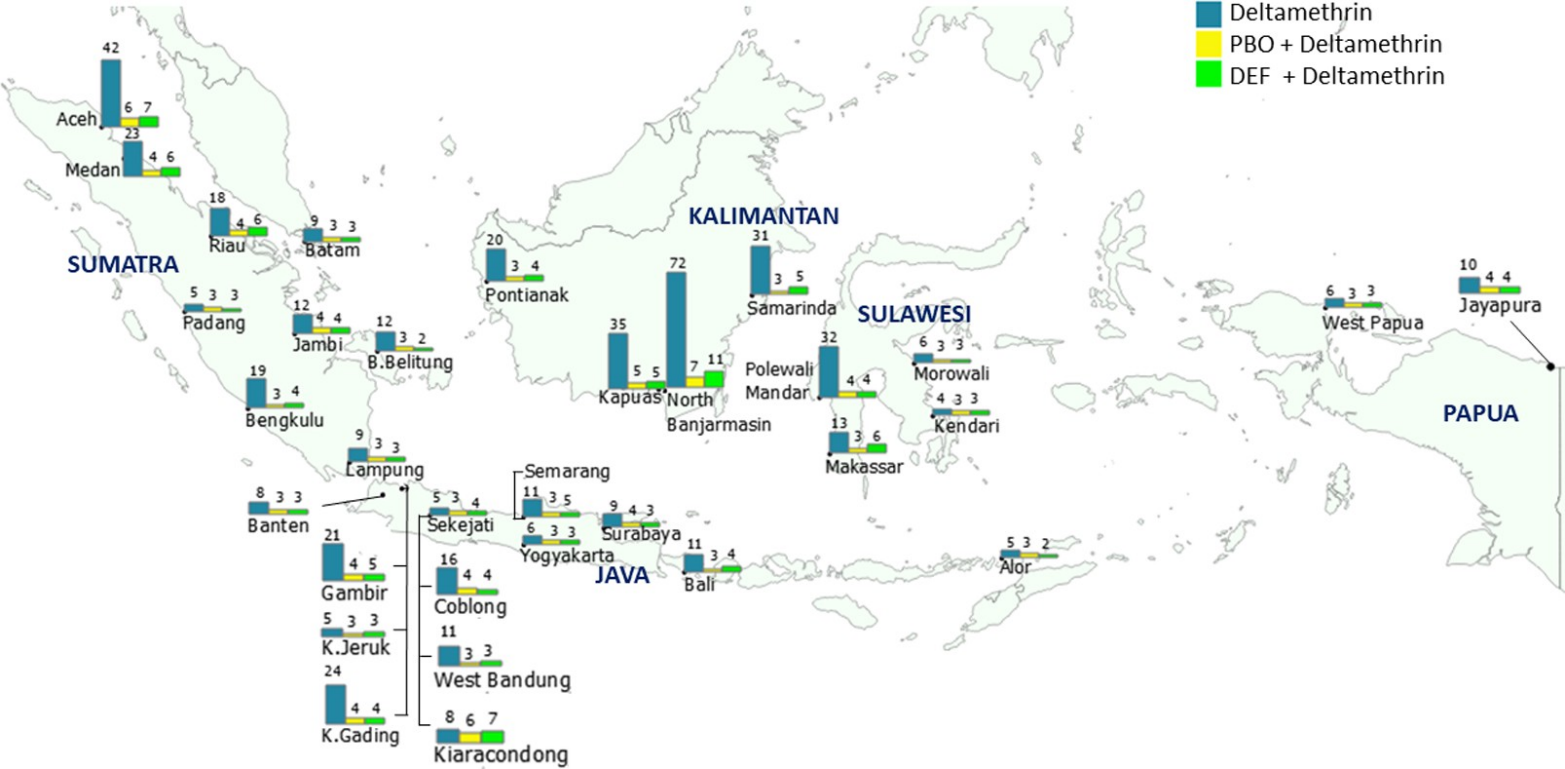
Map of RR_50_ of *Ae*. *aegypti* field strains to deltamethrin without and with synergists. Map base layer was obtained from https://www.naturalearthdata.com/. Map base layer was modified in QGIS software version 3.4 Madeira. Note: The number above each bar represents RR_50_, which was calculated relative to the susceptible reference strain (Bora-Bora).

The RR_50_ of most field strains to deltamethrin decreased to 2.47–7.22× after the PBO treatment, with SR_50_ ranging from 1.25× to 11.71× (Fig 3 and S2 Table). In particular, PBO treatment considerably reduced the resistance of five high-resistance strains of *Ae*. *aegypti* to deltamethrin, namely Samarinda, Polewali Mandar, Kapuas, Aceh, and North Banjarmasin; their resistance levels decreased from 30.76× to 2.69×, 32.12× to 3.75×, 35.29× to 4.53×, 42.06× to 6.16×, and 72.20× to 7.22×, respectively. Most field strains exhibited a considerable decrease in the RR to deltamethrin after DEF exposure, ranging from 2.31× to 10.46× (Fig 3 and S2 Table). The resistance of the five strains with very high resistance to deltamethrin also decreased remarkably after DEF exposure, with SR_50_ ranging from 5.51× to 7.82×. However, of the 32 *Ae*. *aegypti* strains, the deltamethrin resistance of the Kiaracondong strain was unaffected, even after DEF pretreatment; KT_50_ of the deltamethrin treatment alone and the deltamethrin + DEF pretreatment was approximately 69 min.

**Fig 4:**
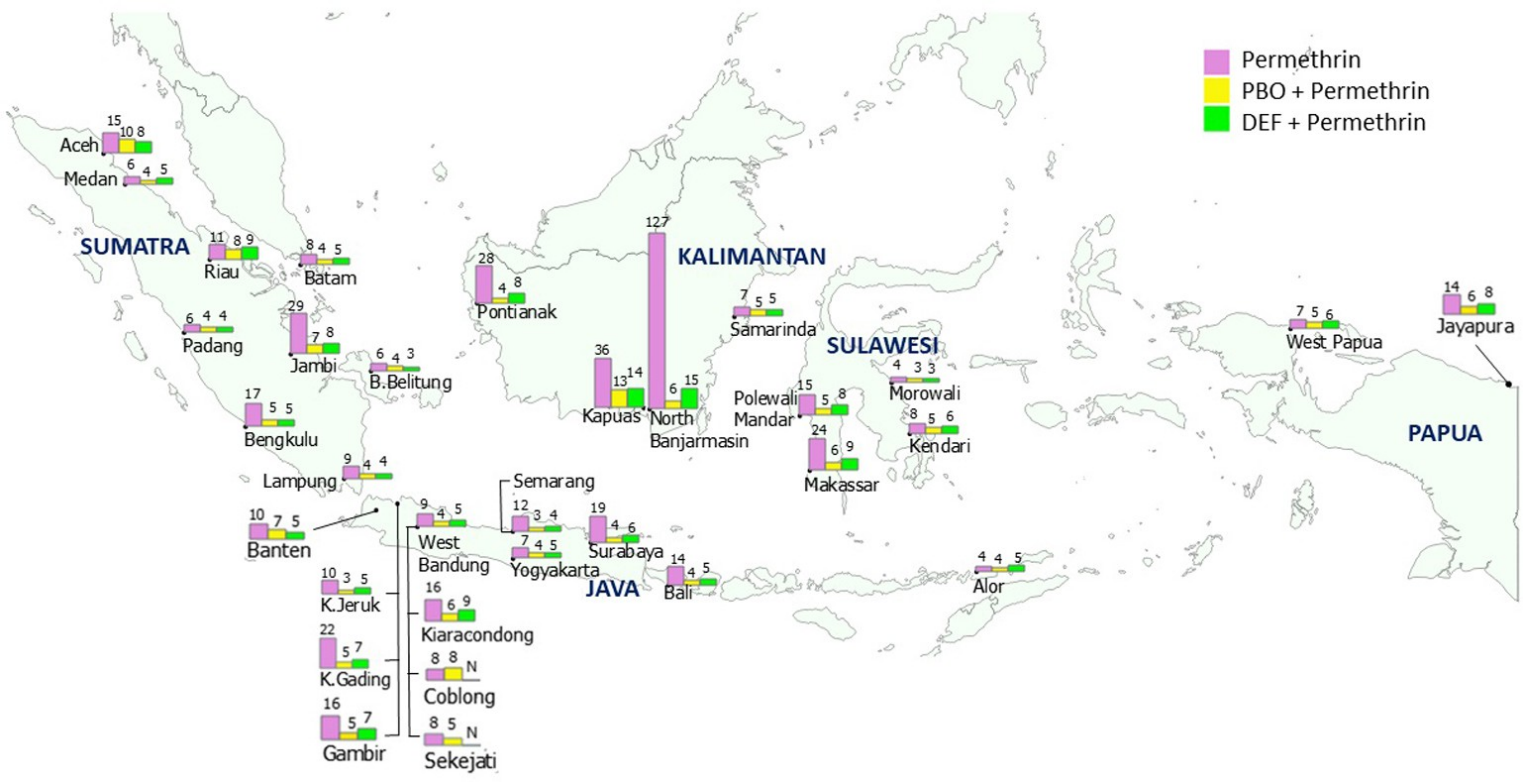
Map of RR_50_ of *Ae*. *aegypti* field strains to permethrin with and without synergists. Map base layer was obtained from https://www.naturalearthdata.com/. Map base layer was modified in QGIS software version 3.4 Madeira. Note: The number above each bar represents RR_50_, which was calculated relative to the susceptible reference strain (Bora-Bora; *N* indicates lack of available data).

Preexposure of the field mosquitoes to PBO or DEF synergists negated permethrin resistance in most field strains, with their RR_50_ values ranging from 3.20× to 13.38× for PBO pretreatment and from 3.30× to 15.29× for DEF pretreatment (Fig 4 and S3 Table). The resistance to permethrin of a very high–resistance strain collected from North Banjarmasin exhibited a substantial decrease from 127.00× to 6.17× after exposure to PBO and from 127.00× to 15.29× after DEF exposure. For a strain from Kapuas with high resistance to permethrin, the resistance decreased to moderate levels after PBO (RR_50_ = 13.38×) and DEF treatment (RR_50_ = 13.70×). By contrast, PBO synergism did not negate permethrin resistance in the Coblong strain (SR_50_ = 1.00×).

## Discussion

*Aedes aegypti* is a container-breeding mosquito because of its affinity for laying eggs in and near standing water. It is well adapted to and can successfully thrive in numerous artificial and natural habitats in urban environments. The reported increase in the occurrence of insecticide resistance and dengue cases was primarily driven by urban expansion and human population growth [17]. For instance, the *Ae*. *aegypti* from the capital city of Indonesia, Jakarta, exhibited low mortality (less than 90%) and high resistance when they were exposed to 0.75% permethrin. The low mortality was linked to *kdr*, which is the V1016G mutation in the VGSC [11]. The presence of the *kdr* mutation has also been reported in the pyrethroid-resistant *Ae*. *aegypti* from Semarang (Central Java), and it resulted in low mortality rates, ranging from 9.3% to 33.3% [18]. Studies have reported multiple *kdr* mutations in the pyrethroid-resistant *Ae*. *aegypti* from Magelang (Central Java) and several areas of Yogyakarta [6,17]. This study selected 11 sites from six major cities on Java Island (i.e., Banten, Jakarta, Bandung, Semarang, Yogyakarta, and Surabaya) for analysis. All *Ae*. *aegypti* populations sampled from Java Island exhibited low to moderate resistance to both deltamethrin and permethrin; relative to that of the laboratory strain, their RR_50_ values were between 5.06× and 24.35× higher for deltamethrin and between 7.27× and 22.33× higher for permethrin. This finding indicates the presence of heterogeneity in the resistance of *Ae*. *aegypti* populations within cities.

Contrary to our expectations, most of the *Ae*. *aegypti* populations collected from Kalimantan, which is a less densely populated island, exhibited high resistance to both deltamethrin and permethrin. The *Ae*. *aegypti* populations from North Banjarmasin (South Kalimantan), Kapuas (Central Kalimantan), and Samarinda (East Kalimantan) exhibited high resistance to deltamethrin. In addition, the North Banjarmasin strain was very highly resistant to permethrin insecticide. This finding is in line with that reported by Hamid et al. [4], who observed a high resistance to 0.75% permethrin and a low mortality rate of less than 50% in the *Ae*. *aegypti* population from Banjarmasin. A proximate explanation for the increased pyrethroid resistance of the *Ae*. *aegypti* populations in Kalimantan is the indirect exposure of *Ae*. *aegypti* to pesticides through agricultural practices. In particular, the rate of conversion of Indonesian forests into oil palm industrial plantations has increased considerably since 2005 [31]. Because of the expansion of palm oil plantations in Kalimantan, agrochemicals, which 40% of the registered insecticides are pyrethroid-based products, are extensively used to manage various insect pests [4]. Thus, the increase in resistance could have occurred in conjunction with the excessive use of pesticides for various purposes, including the control of agricultural pest insects and dengue vectors due to urban sprawl [12,32–34]. Khan et al. [34] conducted a study in Pakistan that revealed various levels of resistance among *Aedes albopictus* populations in cotton fields to several pesticides that have been extensively used to control various cotton pests.

Another non–mutually exclusive explanation is that Kalimantan has reported the highest dengue IR in Indonesia between 2017 and 2019. For instance, North and East Kalimantan had the highest dengue IRs in Indonesia in 2019 [3]. Central and East Kalimantan had the highest dengue IRs in 2018, whereas West Kalimantan had the highest dengue IR in 2017 [1]. The high resistance of *Ae*. *aegypti* to pyrethroids reported in this study is likely associated with the dengue outbreaks in Kalimantan due to the excessive insecticide use during such outbreaks.

Although the field populations from Kalimantan are characterized by high pyrethroid resistance, synergism tests using PBO and DEF significantly reduced the deltamethrin and permethrin resistance in most strains from Kalimantan and other major cities. In addition, the effectiveness of the synergists in negating pyrethroid resistance among the *Ae*. *aegypti* populations collected from Indonesia indicates the possible involvement of metabolic-based resistance mechanisms in most strains of *Ae*. *aegypti*. This finding contradicts that of Koou et al. [26], who reported that the addition of PBO and DEF generally failed to enhance the toxicity of cypermethrin, permethrin, and etofenprox in relation to field populations of *Ae*. *aegypti* mosquitoes from Singapore. However, we did not rule out the involvement of *kdr* resistance and other mechanisms (i.e., elevated glutathione S-transferases, reduced cuticular penetration as exhibited by the malaria vector mosquito, *Anopheles gambiae* [35]) because the pyrethroid resistance of all the collected field populations was not entirely negated by the synergists. Nevertheless, pyrethroids with synergists are still eligible for use in insecticide resistance management plans. Pyrethroids are widely available and well accepted by local authorities in Indonesia in vector control settings.

DEF is an inhibitor of esterases, and esterases are known to confer organophosphate and carbamate resistance to *Aedes* mosquitoes [26,36]. This could explain why government reports have indicated that malathion was ineffective in numerous regions of Indonesia; specifically, the *Ae*. *aegypti* populations in 86 of 102 (84%) tested regencies and cities were demonstrated to be resistant to malathion [37]. By contrast, populations of *Ae*. *aegypti* were discovered to exhibit low levels of resistance to bendiocarb and pirimiphos-methyl at all of the sites surveyed in this study. The susceptibility of field mosquitoes to these insecticides suggests that the cross-resistance among these organophosphate-group insecticides is limited; conversely, for pyrethroids, cross-resistance to deltamethrin and permethrin is well documented and was reported in this study [10,26]. Similar results were observed in specific field populations of *Ae*. *aegypti* from Singapore that exhibited low resistance to pirimiphos-methyl (RR_50_ ranging from 1.01× to 1.51×) [26]. This study demonstrated that bendiocarb and pirimiphos-methyl are effective against all populations tested, making these insecticides potential options for controlling *Ae*. *aegypti* populations in Indonesia. However, a high resistance to bendiocarb was observed in the *Ae*. *aegypti* population in Banjarmasin (South Kalimantan), which had a mortality of less than 50% [4], and in those in Bali (Denpasar) and South Sulawesi (Makassar), which had mortality rates of less than 90% [4,20,21]. Furthermore, the *Ace*-1 gene was detected in the larvae of field strains of *Ae*. *aegypti* from Padang (West Sumatra), which were resistant to temephos (organophosphate group) [38]. Thus, carbamate and organophosphate candidates must be used judiciously.

### Implications and recommendations

Since the 1980s, pyrethroids have been used in numerous applications (including indoor residual spraying [IRS], fogging, and household insecticide products for mosquito control) in Indonesia [6,16,18]. The frequent application of pyrethroids to control *Ae*. *aegypti* increases selection pressure and leads to the development of metabolic resistance and target site insensitivity–linked insecticide resistance [6]. In Indonesia, the implementation of insecticide resistance management at the national level is particularly challenging because Indonesia, which is the largest archipelago in the world, encompasses a land area of 1.919 million km^2^ and exhibits regional disparities in terms of population, health, and socioeconomic status. In Indonesia, information concerning the insecticide resistance profiles of *Ae*. *aegypti* populations is only available for several cities [4,6,22,24,38,10,11,16–21]. The lack of wide-area insecticide resistance surveillance results in fragmentary information. In addition, the heterogeneous response in resistance at the strain level increases the complexity of formulating an appropriate insecticide resistance management strategy at the national level. To complicate matters, the data released by the Ministry of Agriculture of Indonesia in 2022 indicated that approximately 82% of the insecticides (63 out of 76 insecticides) registered for controlling *Ae*. *aegypti* in Indonesia are pyrethroid-based products [39]. Therefore, the available options with respect to insecticide groups for *Aedes* vector management are limited. Consequently, no study has proposed decisive strategies for insecticide resistance management.

The major problem for nationwide insecticide resistance management remains unsolved. This study revealed that the resistance of *Ae*. *aegypti* to pyrethroids (permethrin and deltamethrin) was geographically widespread in Indonesia. In addition, the insecticide resistance of the strains obtained from West Java (West Bandung, Coblong, and Kiaracondong) was moderate to high and similar to that reported in 2006 [10]. This finding suggests that limited progress has been made in terms of the management of the insecticide resistance of *Aedes* vectors in Indonesia. Therefore, the timely implementation of an insecticide resistance management program should be prioritized to prevent the situation from worsening.

At least two insecticide resistance management plans could be implemented. The first is a rotation of deltamethrin (or cyano-group pyrethroids) with synergists, bendiocarb, permethrin (or non–cyano group pyrethroids) with synergists, and pirimiphos-methyl at 6-month intervals; this plan is in compliance with the WHO’s recommendation that insecticides with different modes of action should be rotated on a semiannual basis [40]. A study reported that the yearly rotation of three insecticide classes (organophosphate, carbamate, and pyrethroid) successfully reduced the resistance gene frequencies of the malaria vector *Anopheles albimanus* in southern Mexico [41]. However, whether the 6-month period is optimal before the development of insecticide resistance to the insecticides requires clarification. In addition, the rotation of these insecticides in Indonesia is difficult because most of the registered insecticides for controlling *Ae*. *aegypti* are pyrethroid based; only two are organophosphate based (i.e., pirimiphos-methyl and malathion), and no space-spraying formulation involves the carbamate-group insecticide [39]. A key advance in *Aedes* vectors control is IRS. Several studies have reported highly satisfactory outcomes of the implementation of IRS by using bendiocarb in targeted areas against pyrethroid-resistant strains of *Ae*. *aegypti* in Mexico [42,43]. Our findings underscore the urgent need to approve other non-pyrethroid-based insecticides as alternative tools for reducing the risk of resistance development during an outbreak.

The second feasible strategy for implementing insecticide resistance management programs in Indonesia is the use of insecticide mixtures of pyrethroids and pirimiphos-methyl (organophosphate group) in space-spraying formulations. A combination of pyrethroid and organophosphate insecticides can substantially increase the toxicity of insecticides to specific resistant insect strains, including malaria vector mosquitoes [44], *Musca domestica* [45], and *Helicoverpa armigera* [46]. Mixtures are often used to control malaria vector mosquitoes (clothianidin + deltamethrin [47–49]). However, few mixtures for controlling *Aedes* vector are available. Studies should consider the development of insecticide mixture formulations that can overcome the widespread pyrethroid resistance challenges in Indonesia.

## Supporting information

S1 Table Geographical location of 32 *Ae*. *aegypti* collection sites in Indonesia. (DOCX)

S2 Table Susceptibility of *Ae*. *aegypti* field strains from Indonesia to 0.014% deltamethrin with and without synergists (4% PBO and 5% DEF). (DOCX)

S3 Table Susceptibility of *Ae*. *aegypti* field strains from Indonesia to 0.566% permethrin with and without synergists (4% PBO and 5% DEF). (DOCX)

S4 Table Susceptibility of *Ae*. *aegypti* field strains from Indonesia to 0.212% pirimiphos-methyl. (DOCX)

S5 Table Susceptibility of *Ae*. *aegypti* field strains from Indonesia to 0.1% bendiocarb. (DOCX)
