# Supplementary material for: Insecticide Resistance Profiles and Synergism of Field Aedes aegypti from Indonesia
Source: PLoS Negl Trop Dis. 2022 Jun 6;16(6):e0010501. doi: 10.1371/journal.pntd.0010501 (PMC9203003; doi:10.1371/journal.pntd.0010501)
Supplement: S1 Table — (DOCX) [file pntd.0010501.s001.docx]

S1 Table. Geographical location of 32 *Ae. aegypti* collection sites in Indonesia

| **Island** | **Province** | **Regency/City** | **District** | **Urban Village** | **GPS Coordinates** |
| --- | --- | --- | --- | --- | --- |
| Sumatra | Aceh | Langsa | West Langsa |  | 4°29'14.7"N 97°58'25.1"E |
|  | North Sumatra | Medan | Medan Selayang |  | 3°33'20.8"N 98°37'45.6"E |
|  | West Sumatra | Lubuk Kilangan |  | Bandar Buat | 0°57'07.0"S 100°25'23.4"E |
|  | Riau | Bengkalis | Mandau |  | 1°16'14.7"N 101°12'06.6"E |
|  | Kepulauan Riau | Batam | Sekupang | Tiban Baru | 1°06'20.9"N 103°57'48.0"E |
|  | Jambi | Muaro Jambi | Kumpeh Ulu |  | 1°36'28.1"S 103°40'0.4"E |
|  | Bengkulu | Bengkulu | Singaran Pati | Jembatan Kecil | 3°48'40.5"S 102°17'25.2"E |
|  | Bangka Belitung | Pangkal Pinang | Gerunggang | Bukit Sari | 2°07'13.3"S 106°06'17.6"E |
|  | Lampung |  | Way Halim | Way Halim Permai | 5°23'19.1"S 105°17'10.7"E |
| Java | Banten | Lebak | Rangkasbitung | Cijoropasir | 6º20'38.2"S 106º15'44.9"E |
|  | West Java | West Bandung | Ngamprah |  | 6°51'16.6"S 107°30'50.8"E |
|  |  | Bandung | (1) Coblong | Dago | 6°52'46.0"S 107°36'59.9"E |
|  |  |  | (2) Kiaracondong |  | 6°55'48.5"S 107°39'23.8"E |
|  |  |  | (3) Buahbatu | Sekejati | 6°56'49.02"S 107°39'35.15"E |
|  | Central Java | Semarang | South Semarang | Mugasari | 6°59'30.7"S 110°25'00.7"E |
|  | DKI Jakarta | (1) West Jakarta | Kebon Jeruk | Duri Kepa | 6º10'57.1'S 106º46'36.8"E |
|  |  | (2) Central Jakarta | Gambir | Kebon Kelapa | 6°09'53.4"S 106°49'30.6"E |
|  |  | (3) North Jakarta | Kelapa Gading | Pegangsaan Dua | 6°8'53.8"S 106°54'29.4"E |
|  | DI Yogyakarta | Sleman | Berbah |  | 7°49'13.8"S 110°25'19.9"E |
|  | East Java | Surabaya | Rungkut |  | 7°19'47.6"S 112°47'04.7"E |
| Kalimantan | Central Kalimantan | Kapuas | Tamban Catur |  | 3°14'40.5"S 114°23'55.91"E |
|  | South Kalimantan | North Banjarmasin | North Alalak |  | 3°16'51.9"S 114°34'25.8"E |
|  | West Kalimantan | Pontianak | Southeast Pontianak | Bansir Laut | 0°03'32.1"S 109°19'44.3"E |
|  | East Kalimantan | Samarinda | Samarinda Ulu |  | 0°28'13.39"S 117°9'11.13"E |
| Nusa Tenggara | Bali | Badung | North Kuta |  | 8°37'32.0"S 115°09'19.5"E |
|  | NTT | Alor | Teluk Mutiara | Kalabahi Kota | 8°13'03.3"S 124°30'58.8"E |
| Sulawesi | Central Sulawesi | Morowali | Central Bungku |  | 2°28'41.08"S 121°55'58.72"E |
|  | West Sulawesi | Polewali Mandar | Campalagian | Pappang | 3°28'49.8"S 119°08'29.4"E |
|  | Southeast Sulawesi | Kendari | Baruga | Watubangga | 4°01'34.2"S 122°29'00.0"E |
|  | South Sulawesi | Makassar | Panakkukang |  | 5°08'35.2"S 119°26'39.4"E |
| Papua | Papua | Jayapura | South Jayapura | Entrop | 2°34'12.2"S 140°41'55.4"E |
|  | West Papua | Manokwari |  | Amban | 0°50'12.8"S 134°03'31.7"E |
