# Supplementary material for: Insecticide Resistance Profiles and Synergism of Field Aedes aegypti from Indonesia
Source: PLoS Negl Trop Dis. 2022 Jun 6;16(6):e0010501. doi: 10.1371/journal.pntd.0010501 (PMC9203003; doi:10.1371/journal.pntd.0010501)
Supplement: S2 Table — (DOCX) [file pntd.0010501.s002.docx]

S2 Table. Susceptibility of *Ae. aegypti* field strains from Indonesia to 0.014% deltamethrin with and without synergists (4% PBO and 5% DEF).

| **Strain** | **Deltamethrin** | | | **PBO + Deltamethrin** | | | | **DEF + Deltamethrin** | | | |
| --- | --- | --- | --- | --- | --- | --- | --- | --- | --- | --- | --- |
|  | **KT_50_ (95% FL) (min)** | **Slope** | **RR_50_** | **KT_50_ (95% FL) (min)** | **Slope** | **RR_50_** | **SR_50_** | **KT_50_ (95% FL) (min)** | **Slope** | **RR_50_** | **SR_50_** |
| Bora-Bora | 9.35  (8.68–10.00) | 5.85 ± 0.43 | – | 9.13  (8.79–9.46) | 7.39 ± 0.38 | – | – | 10.39  (9.76–10.98) | 8.06 ± 0.75 | – | – |
| **Aceh** | **393.33**  **(371.37–417.69)** | **2.26 ± 0.10** | **42.06** | 56.19  (54.34–58.07) | 5.01 ± 0.24 | 6.16 | 7.00 | 71.40  (68.31–74.45) | 3.47 ± 0.14 | 6.87 | 5.51 |
| Medan | 211.52  (200.67–222.88) | 3.13 ± 0.12 | 22.62 | 34.13  (33.13–35.14) | 5.77 ± 0.28 | 3.74 | 6.20 | 57.35  (54.85–59.83) | 3.04 ± 0.12 | 5.52 | 3.69 |
| Padang | 47.96  (45.18–50.83) | 3.83 ± 0.16 | 5.13 | 26.34  (25.52–27.13) | 10.46 ± 0.65 | 2.89 | 1.82 | 26.89  (25.04–28.63) | 9.30 ± 0.59 | 2.59 | 1.78 |
| Riau | 164.62  (157.38–172.91) | 4.17 ± 0.21 | 17.60 | 38.25  (37.41–39.09) | 10.37 ± 0.53 | 4.19 | 4.30 | 63.91  (62.13–65.67) | 5.70 ± 0.22 | 6.15 | 2.58 |
| Batam | 81.23  (76.98–85.65) | 3.03 ± 0.15 | 8.69 | 28.61  (27.85–29.33) | 9.58 ± 0.54 | 3.13 | 2.84 | 31.21  (30.03–32.35) | 6.57 ± 0.35 | 3.00 | 2.60 |
| Jambi | 115.55  (103.40–128.16) | 2.34 ± 0.11 | 12.36 | 36.31  (35.35–37.26) | 10.23 ± 0.57 | 3.98 | 3.18 | 42.78  (41.44–44.13) | 7.11 ± 0.41 | 4.12 | 2.70 |
| Bengkulu | 173.19  (157.03–191.53) | 2.57 ± 0.09 | 18.52 | 23.41  (22.60–24.20) | 6.08 ± 0.30 | 2.56 | 7.40 | 36.54  (34.96–38.13) | 5.43 ± 0.30 | 3.52 | 4.74 |
| Bangka Belitung | 111.77  (105.66–119.54) | 3.20 ± 0.12 | 11.95 | 29.07  (27.93–30.18) | 6.25 ± 0.33 | 3.18 | 3.84 | 24.02  (23.08–24.94) | 7.34 ± 0.41 | 2.31 | 4.65 |
| Lampung | 84.54  (77.35–93.32) | 3.01 ± 0.15 | 9.04 | 31.17  (30.22–32.10) | 7.75 ± 0.40 | 3.41 | 2.71 | 29.28  (28.15–30.38) | 6.55 ± 0.34 | 2.82 | 2.89 |
| Banten | 75.53  (71.11–80.53) | 4.04 ± 0.19 | 8.08 | 31.31  (30.37–32.24) | 8.03 ± 0.45 | 3.43 | 2.41 | 33.58  (32.51–34.63) | 7.86 ± 0.42 | 3.23 | 2.25 |
| Gambir | 197.73  (185.26–211.84) | 2.87 ± 0.13 | 21.15 | 36.45  (34.21–38.70) | 5.27 ± 0.28 | 3.99 | 5.43 | 42.07  (40.54–43.62) | 5.21 ± 0.26 | 4.05 | 4.70 |
| Kebon Jeruk | 47.75  (42.77–53.21) | 3.32 ± 0.15 | 5.11 | 24.49  (23.60–25.33) | 7.66 ± 0.38 | 2.68 | 1.95 | 35.06  (33.69–36.46) | 5.33 ± 0.30 | 3.37 | 1.36 |
| Kelapa Gading | 227.67  (211.99–245.24) | 3.04 ± 0.14 | 24.35 | 34.48  (33.28–35.62) | 8.25 ± 0.50 | 3.78 | 6.60 | 42.11  (40.78–43.43) | 6.57 ± 0.32 | 4.05 | 5.41 |

| **Strain** | **Deltamethrin** | | | **PBO + Deltamethrin** | | | | **DEF + Deltamethrin** | | | |
| --- | --- | --- | --- | --- | --- | --- | --- | --- | --- | --- | --- |
|  | **KT_50_ (95% FL) (min)** | **Slope** | **RR_50_** | **KT_50_ (95% FL) (min)** | **Slope** | **RR_50_** | **SR_50_** | **KT_50_ (95% FL) (min)** | **Slope** | **RR_50_** | **SR_50_** |
| West Bandung | 103.29  (97.62–109.59) | 4.46 ± 0.24 | 11.05 | 24.85  (23.21–26.44) | 6.50 ± 0.46 | 2.72 | 4.16 | 29.13  (28.00–30.23) | 6.38 ± 0.33 | 2.80 | 3.55 |
| Kiaracondong | 69.70  (65.17–74.47) | 2.92 ± 0.13 | 7.45 | 55.70  (52.76–58.68) | 4.13 ± 0.14 | 6.10 | 1.25 | 69.61  (66.00–73.14) | 3.82 ± 0.14 | 6.70 | 1.00 |
| Coblong | 151.42  (143.84–159.32) | 3.13 ± 0.15 | 16.19 | 38.67  (37.63–39.67) | 8.32 ± 0.43 | 4.24 | 3.92 | 35.04  (33.73–36.33) | 6.62 ± 0.33 | 3.37 | 4.32 |
| Sekejati | 47.35  (45.86–48.83) | 5.97 ± 0.27 | 5.06 | 27.93  (27.06–28.76) | 10.05 ± 0.60 | 3.06 | 1.70 | 36.38  (33.49–39.14) | 5.46 ± 0.30 | 3.50 | 1.30 |
| Semarang | 102.69  (96.20–110.38) | 3.48 ± 0.18 | 10.98 | 31.27  (30.25–32.22) | 10.10 ± 0.53 | 3.43 | 3.28 | 36.12  (34.15–38.12) | 4.92 ± 0.25 | 3.48 | 2.84 |
| Yogyakarta | 54.93  (48.77–61.21) | 4.18 ± 0.29 | 5.87 | 25.20  (24.18–26.19) | 9.27 ± 0.65 | 2.76 | 2.18 | 29.81  (28.65–30.93) | 8.01 ± 0.49 | 2.87 | 1.84 |
| Surabaya | 80.21  (74.95–85.53) | 3.79 ± 0.20 | 8.58 | 32.80  (31.53–34.04) | 9.12 ± 0.68 | 3.59 | 2.45 | 33.69  (32.64–34.72) | 8.16 ± 0.44 | 3.24 | 2.38 |
| Bali | 106.27  (100.99–112.23) | 3.44 ± 0.16 | 11.36 | 24.68  (24.02–25.33) | 8.25 ± 0.40 | 2.70 | 4.31 | 43.09  (39.81–46.47) | 3.48 ± 0.16 | 4.15 | 2.47 |
| Alor | 46.37  (43.03–50.02) | 5.27 ± 0.27 | 4.96 | 26.06  (24.91–27.15) | 9.54 ± 0.57 | 2.85 | 1.78 | 26.46  (25.41–27.46) | 9.15 ± 0.64 | 2.35 | 1.75 |
| **Kapuas** | **329.96**  **(317.48–343.20)** | **5.26 ± 0.30** | **35.29** | 41.35  (40.07–42.62) | 7.33 ± 0.38 | 4.53 | 7.98 | 50.97  (48.79–59.09) | 5.24 ± 0.21 | 4.90 | 6.47 |
| Pontianak | 190.51  (175.05–209.17) | 2.84 ± 0.11 | 20.37 | 28.00  (26.68–29.18) | 10.31 ± 0.61 | 3.07 | 6.80 | 45.55  (40.83–50.05) | 3.42 ± 0.14 | 4.38 | 4.18 |
| **Samarinda** | **287.62**  **(268.40–308.31)** | **4.01 ± 0.21** | **30.76** | 24.56  (23.37–25.59) | 12.09 ± 0.66 | 2.69 | 11.71 | 52.19  (49.16–55.13) | 4.10 ± 0.14 | 5.02 | 5.51 |
| **North Banjarmasin** | **675.11**  **(655.22–694.58)** | **7.44 ± 0.35** | **72.20** | 65.90  (63.62-68.22) | 4.16 ± 0.17 | 7.22 | 10.24 | 108.77  (103.90–113.80) | 2.99 ± 0.10 | 10.46 | 6.21 |
| **Polewali Mandar** | **300.32**  **(287.34–314.57)** | **4.11 ± 0.23** | **32.12** | 34.21  (32.49–35.88) | 8.28 ± 0.45 | 3.75 | 8.78 | 38.42  (36.33–40.45) | 5.85 ± 0.27 | 3.70 | 7.82 |
| **Strain** | **Deltamethrin** | | | **PBO + Deltamethrin** | | | | **DEF + Deltamethrin** | | | |
|  | **KT_50_ (95% FL) (min)** | **Slope** | **RR_50_** | **KT_50_ (95% FL) (min)** | **Slope** | **RR_50_** | **SR_50_** | **KT_50_ (95% FL) (min)** | **Slope** | **RR_50_** | **SR_50_** |
| Morowali | 57.01  (51.83–62.25) | 4.49 ± 0.27 | 6.10 | 22.58  (21.927–23.223) | 8.04 ± 0.42 | 2.47 | 2.52 | 26.96  (25.74–28.13) | 6.79 ± 0.41 | 2.59 | 2.11 |
| Makassar | 118.93  (112.90–125.48) | 3.88 ± 0.16 | 12.72 | 27.80  (26.93–28.65) | 9.47 ± 0.55 | 3.05 | 4.28 | 61.71  (59.45–63.98) | 3.79 ± 0.15 | 5.94 | 1.93 |
| Kendari | 40.85  (38.96–42.72) | 4.83 ± 0.22 | 4.37 | 25.22  (24.36–26.06) | 9.11 ± 0.60 | 2.76 | 1.62 | 28.82  (27.15–30.46) | 6.55 ± 0.32 | 2.77 | 1.42 |
| Jayapura | 90.95  (86.86–95.45) | 3.87 ± 0.17 | 9.73 | 34.84  (33.74–35.95) | 7.74 ± 0.45 | 3.82 | 2.61 | 44.23  (41.98–46.55) | 5.88 ± 0.30 | 4.25 | 2.06 |
| West Papua | 54.88  (53.18–56.61) | 5.54 ± 0.26 | 5.87 | 29.70  (28.71–30.67) | 8.06 ± 0.43 | 3.25 | 1.85 | 34.23  (33.01–35.41) | 7.86 ± 0.44 | 3.29 | 1.60 |

KT: knockdown time in minute, FL: fiducial limit, RR: resistance ratio, SR: synergist ratio

Strains in bold indicate high resistance or very high resistance to deltamethrin
