# Supplementary material for: Insecticide Resistance Profiles and Synergism of Field Aedes aegypti from Indonesia
Source: PLoS Negl Trop Dis. 2022 Jun 6;16(6):e0010501. doi: 10.1371/journal.pntd.0010501 (PMC9203003; doi:10.1371/journal.pntd.0010501)
Supplement: S3 Table — (DOCX) [file pntd.0010501.s003.docx]

S3 Table. Susceptibility of *Ae. aegypti* field strains from Indonesia to 0.566% permethrin with and without synergists (4% PBO and 5% DEF).

| **Strain** | **Permethrin** | | | **PBO + Permethrin** | | | | **DEF + Permethrin** | | | |
| --- | --- | --- | --- | --- | --- | --- | --- | --- | --- | --- | --- |
|  | **KT_50_ (95% FL) (min)** | **Slope** | **RR_50_** | **KT_50_ (95% FL) (min)** | **Slope** | **RR_50_** | **SR_50_** | **KT_50_ (95% FL) (min)** | **Slope** | **RR_50_** | **SR_50_** |
| Bora-Bora | 8.84  (8.19–9.43) | 5.79 ± 0.47 | – | 8.53  (8.14–8.91) | 7.99 ± 0.50 | – | – | 7.87  (7.46–8.23) | 6.13 ± 0.39 | – | – |
| Aceh | 133.64  (129.41–137.96) | 5.98 ± 0.27 | 15.12 | 88.81  (85.88–91.91) | 5.25 ± 0.21 | 10.42 | 1.50 | 64.98  (63.43–66.54) | 7.94 ± 0.37 | 8.26 | 2.06 |
| Medan | 54.72  (52.82–56.61) | 4.37 ± 0.17 | 6.19 | 30.40  (29.27–31.50) | 6.57 ± 0.35 | 3.57 | 1.80 | 36.12  (34.75–37.48) | 5.47 ± 0.28 | 4.59 | 1.51 |
| Padang | 49.36  (47.87–50.92) | 7.11 ± 0.38 | 5.59 | 34.19  (32.82–35.52) | 8.97 ± 0.51 | 4.01 | 1.44 | 32.03  (31.05–32.99) | 8.94 ± 0.49 | 4.07 | 1.54 |
| Riau | 100.29  (95.56–105.62) | 5.06 ± 0.33 | 11.35 | 68.87  (64.43–73.59) | 5.35 ± 0.21 | 8.08 | 1.46 | 73.42  (71.49–75.37) | 5.64 ± 0.22 | 9.33 | 1.37 |
| Batam | 68.07  (65.51–70.65) | 5.46 ± 0.20 | 7.70 | 31.03  (29.98–32.06) | 7.54 ± 0.40 | 3.64 | 2.19 | 37.96  (36.79–39.12) | 7.62 ± 0.42 | 4.82 | 1.79 |
| Jambi | 259.95  (247.40–273.44) | 3.39 ± 0.17 | 29.42 | 60.84  (58.43–63.19) | 5.97 ± 0.23 | 7.14 | 4.27 | 63.47  (61.94–65.05) | 7.85 ± 0.40 | 8.07 | 4.10 |
| Bengkulu | 146.76  (132.06–163.22) | 1.76 ± 0.08 | 16.61 | 39.54  (38.57–40.51) | 10.83 ± 0.62 | 4.64 | 3.71 | 39.56  (37.71–41.37) | 6.81 ± 0.40 | 5.03 | 3.71 |
| Bangka Belitung | 52.76  (50.06–55.54) | 4.23 ± 0.19 | 5.97 | 33.44  (32.22–34.65) | 7.05 ± 0.44 | 3.92 | 1.58 | 25.95  (24.84–27.03) | 5.84 ± 0.30 | 3.30 | 2.03 |
| Lampung | 82.75  (76.74–89.80) | 4.03 ± 0.19 | 9.36 | 32.99  (32.05–33.91) | 9.74 ± 0.54 | 3.87 | 2.51 | 31.01  (29.86–32.12) | 10.32 ± 0.77 | 3.94 | 2.67 |
| Banten | 88.16  (83.72–92.86) | 4.85 ± 0.23 | 9.98 | 59.85  (58.46–61.33) | 7.04 ± 0.35 | 7.02 | 1.47 | 42.18  (41.03–43.36) | 8.65 ± 0.51 | 5.36 | 2.09 |
| Gambir | 138.19  (131.11–145.70) | 3.37 ± 0.13 | 15.64 | 45.13  (43.77–46.51) | 6.98 ± 0.36 | 5.29 | 3.06 | 58.41  (56.56–60.27) | 4.99 ± 0.22 | 7.42 | 2.37 |
| Kebon Jeruk | 89.02  (80.58–98.28) | 3.14 ± 0.15 | 10.07 | 27.27  (25.87–28.63) | 6.78 ± 0.36 | 3.20 | 3.26 | 35.72  (34.41–37.03) | 5.92 ± 0.33 | 4.54 | 2.49 |
| Kelapa Gading | 197.29  (186.82–208.59) | 3.13 ± 0.14 | 22.33 | 38.66  (37.31–39.98) | 7.30 ± 0.41 | 4.54 | 5.10 | 55.01  (52.60–57.37) | 5.73 ± 0.25 | 6.99 | 3.59 |

| **Strain** | **Permethrin** | | | **PBO + Permethrin** | | | | **DEF + Permethrin** | | | |
| --- | --- | --- | --- | --- | --- | --- | --- | --- | --- | --- | --- |
|  | **KT_50_ (95% FL) (min)** | **Slope** | **RR_50_** | **KT_50_ (95% FL) (min)** | **Slope** | **RR_50_** | **SR_50_** | **KT_50_ (95% FL) (min)** | **Slope** | **RR_50_** | **SR_50_** |
| West Bandung | 81.03  (75.80–86.64) | 3.31 ± 0.16 | 9.17 | 32.29  (38.06–40.54) | 7.21 ± 0.41 | 3.79 | 2.51 | 42.11  (40.71–43.51) | 6.80 ± 0.38 | 5.35 | 1.92 |
| Kiaracondong | 141.95  (137.25–146.84) | 4.97 ± 0.18 | 16.06 | 53.44  (51.58–55.29) | 6.90 ± 0.31 | 6.27 | 2.66 | 68.18  (66.25–70.13) | 5.27 ± 0.21 | 8.67 | 2.08 |
| Coblong | 71.62  (68.43–74.87) | 4.60 ± 0.16 | 8.10 | 71.29  (68.52–74.32) | 6.45 ± 0.37 | 8.36 | 1.00 | No data available | | | |
| Sekejati | 69.81  (67.75–72.08) | 8.93 ± 0.60 | 7.90 | 39.09  (37.69–40.45) | 7.95 ± 0.39 | 4.59 | 1.79 | No data available | | | |
| Semarang | 102.70  (94.59–111.60) | 3.84 ± 0.17 | 11.62 | 28.35  (27.46–29.21) | 6.14 ± 0.30 | 3.33 | 3.62 | 34.40  (33.31–35.48) | 7.71 ± 0.41 | 4.37 | 2.99 |
| Yogyakarta | 64.22  (60.94–67.67) | 6.90 ± 0.48 | 7.27 | 36.87  (35.91–37.82) | 10.31 ± 0.58 | 4.32 | 1.74 | 35.16  (34.18–36.13) | 9.57 ± 0.53 | 4.47 | 1.83 |
| Surabaya | 171.36  (162.98–180.03) | 4.49 ± 0.25 | 19.39 | 34.08  (32.97–35.16) | 11.28 ± 0.80 | 4.00 | 5.03 | 42.95  (41.90–43.99) | 9.96 ± 0.51 | 5.46 | 3.99 |
| Bali | 120.66  (112.35–129.78) | 2.85 ± 0.11 | 13.65 | 34.71  (31.01–38.14) | 8.44 ± 0.47 | 4.07 | 3.48 | 40.20  (38.39–41.96) | 6.69 ± 0.31 | 5.11 | 3.00 |
| Alor | 38.71  (36.03-41.56) | 5.21 ± 0.27 | 4.38 | 29.80  (28.87–30.71) | 9.33 ± 0.54 | 3.50 | 1.30 | 38.32  (37.38–39.24) | 11.35 ± 0.65 | 4.87 | 1.01 |
| **Kapuas** | **313.91**  **(301.36–327.48)** | **4.23 ± 0.23** | **35.52** | 114.02  (107.27–120.75) | 3.53 ± 0.24 | 13.38 | 2.75 | 107.76  (102.12-114.22) | 3.17 ± 0.14 | 13.70 | 2.91 |
| Pontianak | 244.30  (231.30–259.09) | 3.55 ± 0.23 | 27.65 | 34.04  (33.09–34.98) | 9.75 ± 0.53 | 3.99 | 7.18 | 60.85  (59.28–64.42) | 6.83 ± 0.30 | 7.73 | 4.01 |
| Samarinda | 64.09  (52.03–77.87) | 5.90 ± 0.34 | 7.25 | 43.75  (42.59–44.90) | 9.16 ± 0.46 | 5.13 | 1.46 | 42.01  (40.93-43.10) | 9.89 ± 0.60 | 5.34 | 1.53 |
| **North Banjarmasin** | **1122.27**  **(1077.66–1175.00)** | **6.48 ± 0.23** | **127.00** | 52.62  (50.97–54.24) | 7.96 ± 0.36 | 6.17 | 21.33 | 120.30  (115.11-125.53) | 2.31 ± 0.08 | 15.29 | 9.33 |
| Polewali Mandar | 132.31  (125.21–139.81) | 3.89 ± 0.19 | 14.97 | 41.06  (39.64–42.51) | 8.68 ± 0.635 | 4.82 | 3.22 | 61.89  (60.15–63.70) | 6.28 ± 0.31 | 7.87 | 2.14 |
| **Strain** | **Permethrin** | | | **PBO + Permethrin** | | | | **DEF + Permethrin** | | | |
|  | **KT_50_ (95% FL) (min)** | **Slope** | **RR_50_** | **KT_50_ (95% FL) (min)** | **Slope** | **RR_50_** | **SR_50_** | **KT_50_ (95% FL) (min)** | **Slope** | **RR_50_** | **SR_50_** |
| Morowali | 36.08  (35.01–37.14) | 8.33 ± 0.46 | 4.08 | 28.11  (27.17–29.03) | 8.32 ± 0.45 | 3.30 | 1.28 | 26.64  (25.40–27.84) | 5.12 ± 0.27 | 3.39 | 1.35 |
| Makassar | 207.96  (198.48–218.18) | 3.00 ± 0.11 | 23.53 | 52.71  (50.64–54.75) | 5.93 ± 0.24 | 6.18 | 3.95 | 67.95  (61.65–74.02) | 2.49 ± 0.07 | 8.64 | 3.06 |
| Kendari | 67.79  (64.54–71.18) | 4.57 ± 0.18 | 7.67 | 38.80  (37.57–40.05) | 7.36 ± 0.43 | 4.55 | 1.75 | 46.25  (44.99–47.53) | 7.92 ± 0.42 | 5.88 | 1.47 |
| Jayapura | 126.37  (120.92–132.04) | 5.12 ± 0.22 | 14.30 | 46.78  (45.63–47.93) | 9.41 ± 0.50 | 5.49 | 2.70 | 60.01  (58.62–61.40) | 8.45 ± 0.40 | 7.63 | 2.11 |
| West Papua | 57.13  (55.52–58.74) | 6.12 ± 0.26 | 6.46 | 38.76  (36.86–40.60) | 7.19 ± 0.37 | 4.55 | 1.47 | 46.29  (44.70–47.86) | 7.81 ± 0.47 | 5.88 | 1.23 |

KT: knockdown time in minute, FL: fiducial limit, RR: resistance ratio, SR: synergist ratio

Strains in bold indicate high resistance or very high resistance to permethrin
