# Supplementary material for: Insecticide Resistance Profiles and Synergism of Field Aedes aegypti from Indonesia
Source: PLoS Negl Trop Dis. 2022 Jun 6;16(6):e0010501. doi: 10.1371/journal.pntd.0010501 (PMC9203003; doi:10.1371/journal.pntd.0010501)
Supplement: S4 Table — (DOCX) [file pntd.0010501.s004.docx]

S4 Table. Susceptibility of *Ae. aegypti* field strains from Indonesia to 0.212% pirimiphos-methyl

| **Strain** | **KT_50_ (95% FL) (min)** | **Slope** | **RR_50_** |
| --- | --- | --- | --- |
| Bora-Bora | 168.60 (164.59–172.52) | 10.94 ± 0.64 | - |
| Aceh | 390.61 (385.57–395.66) | 13.86 ± 0.56 | 2.32 |
| Medan | 282.90 (276.96–288.76) | 10.45 ± 0.63 | 1.68 |
| Padang | 453.56 (430.22–485.06) | 6.57 ± 0.46 | 2.69 |
| Riau | 287.56 (282.26–293.00) | 9.00 ± 0.41 | 1.71 |
| Batam | 182.39 (178.75–186.01) | 10.86 ± 0.54 | 1.08 |
| Jambi | 268.04 (263.82–272.23) | 13.29 ± 0.70 | 1.59 |
| Bengkulu | 340.23 (332.77–347.71) | 10.16 ± 0.54 | 2.02 |
| Bangka Belitung | 397.56 (389.02–406.49) | 9.91 ± 0.57 | 2.36 |
| Lampung | 278.94 (272.69–285.26) | 10.45 ± 0.68 | 1.65 |
| Banten | 281.38 (274.51–288.42) | 8.91 ± 0.51 | 1.67 |
| Gambir | 285.83 (281.72–289.84) | 14.48 ± 0.67 | 1.70 |
| Kebon Jeruk | 368.93 (362.81–375.28) | 10.02 ± 0.51 | 2.19 |
| Kelapa Gading | 310.29 (303.36–317.60) | 9.51 ± 0.53 | 1.84 |
| West Bandung | 412.44 (403.99–421.83) | 9.85 ± 0.67 | 2.45 |
| Kiaracondong | 441.79 (425.10–462.72) | 6.59 ± 0.50 | 2.62 |
| Coblong | 273.03 (268.41–277.47) | 13.56 ± 0.68 | 1.62 |
| Sekejati | 454.49 (434.91–481.46) | 6.56 ± 0.45 | 2.70 |
| Semarang | 300.27 (294.13–306.84) | 10.33 ± 0.72 | 1.78 |
| Yogyakarta | 348.11 (337.42–360.16) | 10.10 ± 0.71 | 2.06 |
| Surabaya | 181.41 (178.40–184.44) | 15.67 ± 0.87 | 1.08 |
| Bali | 272.13 (266.59–277.83) | 10.49 ± 0.72 | 1.61 |
| Alor | 272.36 (265.29–280.05) | 10.48 ± 0.80 | 1.62 |
| Kapuas | 285.94 (280.03–292.44) | 10.40 ± 0.77 | 1.70 |
| Pontianak | 303.87 (295.71–312.08) | 10.32 ± 0.67 | 1.80 |
| Samarinda | 195.44 (190.17–200.46) | 8.70 ± 0.47 | 1.16 |
| North Banjarmasin | 305.05 (300.43–309.50) | 13.78 ± 0.67 | 1.81 |
| Polewali Mandar | 170.66 (165.87–175.34) | 10.03 ± 0.68 | 1.01 |
| Morowali | 260.56 (255.87–265.21) | 9.84 ± 0.42 | 1.55 |
| Makassar | 295.11 (288.43–302.63) | 10.35 ± 0.67 | 1.75 |
| **Strain** | **KT_50_ (95% FL) (min)** | **Slope** | **RR_50_** |
| Kendari | 421.08 (411.81–431.26) | 9.82 ± 0.61 | 2.50 |
| Jayapura | 244.87 (239.60–250.14) | 10.68 ± 0.65 | 1.45 |
| West Papua | 268.48 (263.36–273.31) | 15.54 ± 0.81 | 1.59 |

KT: knockdown time in minute, FL: fiducial limit, RR: resistance ratio
