# Supplementary material for: Insecticide Resistance Profiles and Synergism of Field Aedes aegypti from Indonesia
Source: PLoS Negl Trop Dis. 2022 Jun 6;16(6):e0010501. doi: 10.1371/journal.pntd.0010501 (PMC9203003; doi:10.1371/journal.pntd.0010501)
Supplement: S5 Table — (DOCX) [file pntd.0010501.s005.docx]

S5 Table. Susceptibility of *Ae. aegypti* field strains from Indonesia to 0.1% bendiocarb

| **Strain** | **KT_50_ (95% FL) (min)** | **Slope** | **RR_50_** |
| --- | --- | --- | --- |
| Bora-Bora | 16.79 (15.97–17.58) | 6.90 ± 0.47 | - |
| Aceh | 40.92 (39.94–41.87) | 11.75 ± 0.70 | 2.44 |
| Medan | 32.49 (31.84–33.14) | 10.15 ± 0.56 | 1.93 |
| Padang | 31.59 (30.50–32.65) | 11.57 ± 0.90 | 1.88 |
| Riau | 30.23 (28.96–31.32) | 11.90 ± 0.71 | 1.80 |
| Batam | 35.69 (34.75–36.58) | 9.17 ± 0.53 | 2.13 |
| Jambi | 28.74 (28.06–29.39) | 10.95 ± 0.69 | 1.71 |
| Bengkulu | 31.96 (30.98–32.90) | 8.76 ± 0.46 | 1.90 |
| Bangka Belitung | 28.10 (27.04–29.13) | 10.58 ± 0.80 | 1.67 |
| Lampung | 28.56 (27.61–29.50) | 8.92 ± 0.56 | 1.70 |
| Banten | 29.52 (28.59–29.72) | 12.26 ± 0.68 | 1.76 |
| Gambir | 36.41 (35.37–37.48) | 8.83 ± 0.40 | 2.17 |
| Kebon Jeruk | 27.25 (26.06–28.41) | 8.42 ± 0.55 | 1.62 |
| Kelapa Gading | 108.40 (102.48–114.88) | 3.71 ± 0.19 | 6.46 |
| West Bandung | 33.76 (33.12–34.37) | 13.79 ± 0.79 | 2.01 |
| Kiaracondong | 38.10 (36.99-39.21) | 8.29 ± 0.47 | 2.27 |
| Coblong | 30.84 (29.94-31.71) | 10.28 ± 0.61 | 1.84 |
| Sekejati | 36.84 (35.76–37.88) | 8.78 ± 0.44 | 2.19 |
| Semarang | 44.03 (42.67–45.41) | 6.67 ± 0.35 | 2.62 |
| Yogyakarta | 33.11 (31.75–34.43) | 8.47 ± 0.45 | 1.97 |
| Surabaya | 45.73 (44.51–47.02) | 9.26 ± 0.60 | 2.72 |
| Bali | 78.03 (75.81–80.45) | 6.26 ± 0.31 | 4.65 |
| Alor | 21.16 (20.52–21.76) | 12.66 ± 0.81 | 1.26 |
| Kapuas | 20.79 (19.91–21.64) | 7.22 ± 0.44 | 1.24 |
| Pontianak | 35.51 (34.42–36.65) | 8.57 ± 0.59 | 2.11 |
| Samarinda | 38.83 (37.73–39.93) | 9.16 ± 0.54 | 2.31 |
| North Banjarmasin | 52.68 (51.68–53.70) | 8.91 ± 0.43 | 3.14 |
| Polewali Mandar | 24.71 (24.24–25.16) | 15.89 ± 1.02 | 1.47 |
| Morowali | 26.88 (26.21–27.51) | 11.31 ± 0.68 | 1.60 |
| Makassar | 32.40 (31.58–33.21) | 12.52 ± 0.77 | 1.93 |
| **Strain** | **KT_50_ (95% FL) (min)** | **Slope** | **RR_50_** |
| Kendari | 30.05 (29.33–30.77) | 9.32 ± 0.51 | 1.79 |
| Jayapura | 37.15 (36.33–37.95) | 8.16 ± 0.35 | 2.21 |
| West Papua | 35.83 (35.09–36.57) | 9.44 ± 0.52 | 2.13 |

KT: knockdown time in minute, FL: fiducial limit, RR: resistance ratio
